# The association between adverse pregnancy outcomes with genital *Chlamydia Trachomatis* infection among pre-pregnancy couples in Shenzhen, China: A cross-sectional study

**DOI:** 10.3389/fpubh.2022.1038391

**Published:** 2022-12-09

**Authors:** Si Sun, Li Zhang, Qiuhong Wu, Lishan Tian, Yi Ding, Lanlan Liu, Hailing Ye, Bo Li, Zhenzhou Luo

**Affiliations:** Shenzhen Nanshan Center for Chronic Disease Control, Shenzhen, China

**Keywords:** *Chlamydia Trachomatis*, GCT, adverse pregnancy outcome, APOs, concordant

## Abstract

**Objectives:**

To investigate the prevalence of adverse pregnancy outcomes (APOs) in women and the impact of pre-pregnancy couples' genital *Chlamydia Trachomatis* (GCT) infection and other infections on APOs.

**Study design:**

Data on genital infections were collected from the Free Pre-pregnancy Health Check (FPHC) in Shenzhen, China. Data on APOs were collected from a 1-year telephone follow-up of pregnancy status and subsequent pregnancy outcomes.

**Methods:**

APO data were used to count adverse outcomes, and logistic regression was conducted to determine the association between APOs and GCT infection.

**Results:**

From December 2018 to December 2019, among 4,429 couples who underwent FPHC; 1,925 were pregnant, and 1,816 couples were tracked for pregnancy outcomes, including 1,471 normal pregnancies and 345 (19.00%) APOs. The rest of 109 pregnant couples did not answer the phone or refused to answer the pregnancy outcome during the follow-up. Among APOs, the number of spontaneous abortions was 122 (35.36%), the number of macrosomia was 85 (24.64%), the number of low birth weight (LBW) & preterm births (PTB) was 39 (11.30%), the number of LBW was 34 (9.86%), and the number of PTB was 31 (8.99%). The prevalence of GCT infection in females and males was 4.24% [95% Confidence Interval, (CI): 3.41–5.27%] and 3.58% (95% CI: 2.79–4.57%), respectively. More than half (52.69%, 49/93) of the couples were GCT-concordant. The prevalence of APOs in couples without GCT infection was 18.74% (332/1,772). The prevalence of APOs in female GCT-discordant was 32.14% (9/28), and the prevalence of APOs in male GCT-discordant was 25% (4/16). The prevalence of APOs in GCT-concordant was 12.24% (6/49). Multivariable analysis indicated that females 30–35 years old [adjusted Odds Ratio (aOR) = 1.08, 95% CI: 1.01–1.17] and over 35 years old (aOR = 1.16, 95% CI: 1.03–1.32) were more likely to experiencing APOs.

**Conclusion:**

Although only women's age was found to be associated with APOs, the prevalence of APOs with GCT-discordant in couples, especially female GCT-discordant, was higher than in those without infection or who were GCT-concordant, suggesting that these groups, especially in older women, should be paid more attention to in follow-ups to improve reproductive health.

## Background

Adverse pregnancy outcomes (APOs) are important public health issues, mainly including spontaneous abortion (SA), stillbirth, ectopic pregnancy, preterm births (PTB), low birth weight (LBW), macrosomia, birth defects, etc., (1). APOs are harmful to the health of pregnant women and fetuses and increase the risk of chronic non-communicable diseases such as adult obesity, hypertension, and diabetes in offspring (2–4). APOs seriously affect the economy and spirit of relevant families.

The occurrence of APOs is related to many factors. Genital *Chlamydia Trachomatis* (GCT) infection is one of the world's most common sexually transmitted infections (5). And studies have shown that GCT infection is related to the occurrence and development of many APOs (1). In 2011, Johnson et al. (6) found that GCT infection significantly increased the risk of LBW in newborns (aOR: 2.07, 95% CI: 1.01–4.24) in the United States (US). A case-control study was done in October 2013 through June 2014 in Iran showed the prevalence of GCT infection in pregnant women with a history of SA was significantly higher than that in pregnant women with normal childbirth (7), and *Chlamydia Trachomatis* (CT) DNA was more common in the pregnancy products and placenta of aborted women (8). In 2018, a meta-analysis showed that there was a slight but statistically significant overall association between GCT infection and PTB (OR = 1.27, 95% CI: 1.05–1.54) (9). In 2020, a systematic review showed that mothers with GCT infection were 1.35 times (OR = 1.35, 95% CI: 1.03–1.76) more likely to develop adverse outcomes than non-infected mothers while reducing GCT infection significantly improved pregnancy outcomes (OR = 0.43; 95% CI: 0.27–0.68) (10).

Screening and treatment of GCT infection can help reduce APOs. For instance, early screening and treatment of GCT infection would significantly reduce the risk of PTB in pregnant women (11). However, since about 70% of women and 50% of men with GCT infection are asymptomatic (12), active screening of high-risk groups is an effective way to prevent and treat GCT infection (13). Currently, countries mainly screen women for GCT infection (12, 14, 15). Still, some studies show that the concordant rate of GCT infection between male and female sexual partners is 10–75%, which means that some women may still be threatened by GCT infection if the infections of these women's partners are not diagnosed and treated timely (16). However, there's no study on the impact of the GCT-concordant status on APOs in China. So, this study was conducted to explore the impact of the GCT infection, the GCT-concordant status of pre-pregnancy couples, and other factors on APOs, through long-term follow-up, and to provide ideas for GCT infection and APOs prevention strategies.

## Method

### Study participants

Study participants were couples participated in the Free Pre-pregnancy Health Check (FPHC) in Nanshan District, Shenzhen from December 2018 to December 2019. Participants were eligible for participation if they met the following inclusion criteria: (1) willing to test GCT by nucleic acid detection method; (2) willing to participate and signed the informed consent. Before the analysis, the records of all couples were anonymous. This study was an observational study, which was beneficial and harmless to the subjects.

### Data collection

The medical staff followed up on the pregnancy status of the couples who participated in the FPHC by telephone 1 year after the FPHC. If they were pregnant within 1 year, we would continue to follow up on their pregnancy outcomes by inquiring about couples. If they were not pregnant within 1 year, the follow-up would be terminated. Follow-up pregnancy outcomes included normal pregnancy, SA, induced abortion in the medical department, therapeutic induced labor, stillbirth, PTB, LBW, macrosomia, and ectopic pregnancy.

The APOs in the current study included (17) (1) SA defined as fetal death occurring before 28 weeks of gestation; (2) PTB (delivery at a gestational age between 28 and <37 weeks); (3) macrosomia (newborn birth weight ≧4,000 g); (4) LBW (newborn birth weight <2,500 g); (5) stillbirth (intrauterine death of the fetus after 20 weeks of pregnancy); (6) induced abortion in the medical department (pregnancy termination by medical methods due to diseases and other reasons within 14 weeks of pregnancy); (7) therapeutic induced labor (pregnancy termination by medical methods due to diseases and other reasons after more than 14 weeks); and (8) ectopic pregnancy (the embryo attaches outside the uterus).

### Female vagina swab samples test

After the medical staff inserted the vaginal dilator to dilate the vagina, the medical staff used a sterile cotton swab to collect the secretion in the posterior vault of the vagina. Medical staff then performed a smear or dye microscopic examination for genital *Candida*, trichomonas, clue cells, pH, whiff test, and vaginal cleanliness. According to Amsel's criteria (18) and other research (19), two of three criteria, including clue cells, pH, and whiff test, been presented to confirm bacterial vaginosis (BV) diagnosis.

### Female and male urine samples test

The participants held urine for 2 h, collected 10–20 ml of fresh anterior urine, and then staff transferred 3–5 ml urine to a Roche Cobas urine collection tube (Roche/n05170486190). Samples were stored in a 4°C environment and detection of CT and *Neisseria gonorrhoeae* (NG) by Roche cobas 4800 system occurred within 24 h according to the instruction manual (20). The rest of the urine was tested for proteinuria, occult blood, and white blood cells within 1 h.

Wives with GCT infections whose husband did not have a GCT infection were considered female discordant (female GCT-discordant). Conversely, husbands with GCT infections whose wives did not have a GCT infection were considered male discordant (male GCT-discordant). Wives and husbands with GCT infections were considered concordant (GCT-concordant) (16).

### Statistical analysis

Two staff members entered all follow-up information into Epidata 3.0 software (Epidata Association from Denmark), and all test data were from their outpatient records. The test data was exported to Excel software through the outpatient system, and then the follow-up data and test data were matched through the medical registration number. The Chi-square or Fisher test was used to compare the categorical variables between groups, and variables with *P* < 0.2 were incorporated into the univariate and multivariable logistic regressions. In the multivariable model, we adjusted for female age, female proteinuria, male age, and GCT-concordant status. We reported odds ratios (*OR*), 95% confidence intervals (CI) and *P*-values. Results are deemed to be statistically significant when *P* ≤ 0.05. All analyses were conducted on R software 3.6.1 (R Development Core Team, Vienna, Austria).

## Results

### Sociodemographic characteristics

Overall, 4,429 couples participated in the Free Pre-pregnancy Health Check (FPHC), of which 1,925 couples were pregnant, and 1,816 couples were followed up for pregnancy outcomes. The average age of females among the 1,816 couples was 28.13 ± 3.16 years old, and the average age of males was 29.5 ± 3.6.

### Prevalence of APOs

**Figure 1:**
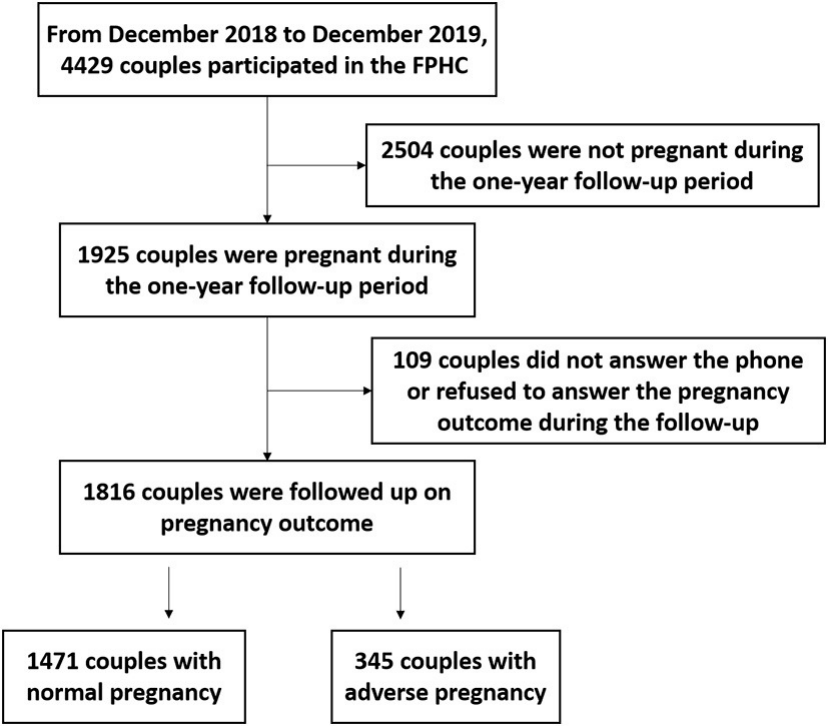
Flowchart of follow-up process on pregnancy outcomes. FPHC, Free Pre-pregnancy Health Check.

During the 1-year follow-up period, 1,925 couples were pregnant, 1,816 were followed up on pregnancy outcomes, and 345 (19.00%) cases of APOs were found (Figure 1). Among the APOs, the majority were SA, macrosomia, and PTB combined with LBW, accounting for 35.36% (prevalence 6.72%), 24.64% (4.68%), and 11.30% (2.15%), respectively (Table 1).

**Table 1:** APOs and general characteristics of pregnant couples (*n* = 1,816).

**Characteristics**	**Number of cases**	**Prevalence (%)**	**Composition ratio (%)**
APOs	345	19.00	100
SA	122	6.72	35.36
Macrosomia	85	4.68	24.64
PTB & LBW	39	2.15	11.30
LBW	34	1.87	9.86
PTB	31	1.71	8.99
Stillbirth	10	0.55	2.90
Therapeutic induced labor	9	0.50	2.61
Ectopic pregnancy	8	0.44	2.32
Induced abortion in medicinedepartment	7	0.39	2.03
**Female**			
Candida	210	11.56	-
BV	53	2.92	-
Vaginal cleanliness III	1,137	62.61	-
Vaginal cleanliness IV	350	19.27	-
Urine occult blood	246	13.55	-
Proteinuria	90	4.96	-
Urine white blood cells	289	15.91	-
GCT	77	4.24	-
**Male**			
Urine occult blood	139	7.65	-
Proteinuria	114	6.28	-
Urine white blood cells	40	2.20	-
GCT	65	3.58	-
**GCT-concordant status**			
Female GCT-discordant	28	1.54	-
Male GCT-discordant	16	0.88	-
GCT-concordant	49	2.70	-

### Prevalence of GCT and other infections

The detection of female swab samples found that the vaginal cleanliness of most females (81.88%) reached or exceeded grade III, and 11.56% of females had *Candida* infection. Urine samples showed that 13.55% of females had urine occult blood, 4.96% of females had proteinuria, 15.91% of females had urine leukocytes, 7.65% of males had urine occult blood, 6.28% of males had proteinuria, and 2.20% of males had urine leukocytes. The nucleic acid test results of GCT infection in the reproductive tract showed that the prevalence of GCT infection in females and males were 4.24% (95% CI: 3.41–5.27%) and 3.58% (95% CI: 2.79–4.57%), respectively. More than half (52.69%, 49/93) of the couples were GCT-concordant, 31.11% of the couples were female GCT-discordant, and 17.20% of the couples were male GCT-discordant (Table 1). *Trichomonas* and NG were not found in all participants.

### Factors correlated with APOs

Chi-square or Fisher test results showed that except for the couple's age, which was related to APOs, no relationship between GCT infection and other symptoms and APOs was observed (Table 2). In the multivariable logistic analysis, female 30–35 years old [adjusted Odds Ratio (aOR) = 1.08, 95% CI: 1.01–1.17] and over 35 years old (aOR = 1.16, 95% CI: 1.03–1.32) were more likely to experiencing APOs (Table 3). Still, the data showed that the prevalence of APOs in female GCT-discordant and male GCT-discordant were higher than the prevalence in non-infected couples (Table 2), even though female GCT-discordant (aOR = 1.13, 95% CI: 0.97–1.30), male GCT-discordant (aOR = 1.05, 95% CI: 0.87–1.28) and GCT-concordant (aOR = 0.95, 95% CI: 0.85–1.06) were not associated with APOs (Table 3).

**Table 2:** Chi-square test for APOs and couples' infections or symptoms.

**Characteristics**	**Normal pregnancy**	**APOs**	**% of APOs**	**χ2**	***P***
**Female**					
**Age (years)**				21.73	<0.001
20–25	160	23	12.57		
26–30	935	199	17.55		
30–35	330	99	23.08		
>35	46	24	34.29		
**Candida**				<0.01	1.000
No	1,301	305	18.99		
Yes	170	40	19.05		
BV				0.04	0.839
No	1,427	336	19.06		
Yes	44	9	16.98		
**Vaginal cleanliness**				2.85	0.415
I	235	59	20.07		
II	26	9	25.71		
III	933	204	17.94		
IV	277	73	20.86		
**Urine occult blood**				0.32	0.572
No	1,268	302	19.24		
Yes	203	43	17.48		
**Proteinuria**				3.31	0.069
No	1,391	335	19.41		
Yes	80	10	11.11		
**Urine white blood cells**				<0.01	0.947
No	1,236	291	19.06		
Yes	235	54	18.69		
**CT**				<0.01	1.000
No	1,409	330	18.98		
Yes	62	15	19.48		
**Male**					
**Age (years)**				16.76	0.001
22–25	78	10	11.36		
26–30	768	164	17.6		
30–35	524	127	19.51		
>35	101	44	30.34		
**Urine occult blood**				<0.01	1.000
No	1,358	319	19.02		
Yes	113	26	18.71		
**Proteinuria**				<0.01	1.000
No	1,379	323	18.98		
Yes	92	22	19.30		
**Urine white blood cells**				<0.01	1.000
No	1,439	337	18.98		
Yes	32	8	20.00		
**CT**				0.35	0.552
No	1,416	335	19.13		
Yes	55	10	15.38		
**CT-concordant status**				—	0.165*
Non-infected couples	1,397	326	18.92		
Female GCT-discordant	19	9	32.14		
Male GCT-discordant	12	4	25.00		
GCT-concordant	43	6	12.24		

*Fisher test.

**Table 3:** Univariate and multivariable logistic analysis of factors associated with APOs.

**Characteristics**	**Crude model**		Adjusted model*	
	**OR (95% CI)**	***P***	**aOR (95% CI)**	***P***
**Female**
Age (years)				
20–25	Ref	-	Ref	-
26–30	1.05 (0.99–1.12)	0.109	1.04 (0.97–1.11)	0.286
30–35	1.11 (1.04–1.19)	0.002	1.08 (1.01–1.17)	0.036
>35	1.24 (1.12–1.38)	<0.001	1.16 (1.03–1.32)	0.018
Proteinuria				
No	Ref	-	Ref	-
Yes	0.92 (0.85–1.00)	0.050	0.93 (0.86–1.01)	0.087
**Male**				
Age (years)				
22–25	Ref	-	Ref	-
26–30	1.06 (0.98–1.16)	0.153	1.05 (0.96–1.14)	0.327
30–35	1.08 (0.99–1.18)	0.067	1.04 (0.95–1.15)	0.360
>35	1.21 (1.09–1.34)	<0.001	1.11 (0.99–1.25)	0.068
**GCT-concordant status**				
Non-infected couples	Ref	-	Ref	*-*
Female GCT-discordant	1.14 (0.99–1.32)	0.077	1.13 (0.97–1.30)	0.111
Male GCT-discordant	1.06 (0.88–1.29)	0.537	1.05 (0.87–1.28)	0.592
GCT-concordant	0.94 (0.84–1.05)	0.240	0.95 (0.85–1.06)	0.339

^*^Female age, female proteinuria, male age, and GCT-concordant status were adjusted for each other.

## Discussion

A cross-sectional study conducted in Nanshan, Shenzhen, from December 2018 to December 2019 found that the prevalence of APOs in pre-pregnancy couples in this region was 19.00%, which was similar to the prevalence (15.60%) in other areas of China (17). In this study, the highest of APOs' constituent ratio was with SA (35.36%), which was higher than that in other regions (18.94%) (21). This may be related to the high work pressure of Shenzhen residents, resulting in high psychological stress, and increasing the prevalence of SA (22). The prevalence of LBW (4.02%) was similar to that of the 2010 study in Shaanxi Province (4.4%), while the prevalence of macrosomia (4.68%) was slightly lower (6.3% for macrosomia) than the same 2010 study (23). The prevalence of preterm birth (3.86%) was much lower than that reported by a survey covering 132 cities in China from 2010 to 2013 (24).

It is worth noting that this study found that the prevalence of SA, macrosomia, LBW, and PTB was high in all APOs. In contrast, the prevalence of stillbirth, induced abortion in medicine department, and therapeutic induced labor was low; this situation was similar to other research (25). However, we should be aware that PTB, LBW, and macrosomia are related to chronic diseases in children, such as neurodevelopmental disorders, cardiovascular diseases, and metabolic diseases (26, 27). In addition, PTB-related complications are closely related to neonatal death (27). Therefore, close attention should be paid to these three APOs and effective public health measures should be taken.

This study found that female age was the influencing factor of APOs. With an increase in female age, the incidence of APOs also increased, which is consistent with Frederiksen et al. (28). A Swiss study involving 2,009,068 pregnant females showed that old age was a significant risk factor for PTB, especially for extreme PTB at 22–31 weeks (29). A multicenter study in the UK showed that the risk of premature delivery among older pregnant females increased by 2.5 times (30). This association likely due to placental dysfunction mediate APOs in advanced maternal age (31).

The prevalence of GCT infection in females (4.24%) in Shenzhen was similar to our previous research (4.12%) (32) and the prevalence in females in the WHO Western Pacific region (4.3%). The prevalence of GCT infection in males (3.58%) in Shenzhen was similar to that of males in the WHO Western Pacific region (3.4%) (5). We did not find an association between male or female GCT infection and APOs, which was expected because all patients were informed of their infection through messages and advised to treat. Vercruysse et al. (33) reported that urogenital CT infection in pregnancy, if adequately treated, has nothing to do with PTB. However, since we could not obtain the treatment data of all patients, we could not accurately answer the association between GCT infection and APOs. Still, meta-analysis showed that in the unadjusted analysis, GCT infection was related to the increased risk of APOs (1).

There were few GCT concordant status studies on GCT infection (16, 34, 35) which may help to understand the extent of sexual partners' infection with the same pathogen. If couples did not test for CT, female or male partners are prone to misdiagnosis, as this omission may lead to GCT infection. In this study, half (52.69%) of the couples with positive GCT infection results were concordant. Similarly, Quinn et al. (35) reported 52% concordant in 101 CT positive samples, Guerra-Infante et al. (36) reported 57% concordant in 14 positive samples, and Schillinger et al. (34) reported 55% concordant in 128 positive samples. Although we did not find an association between GCT-discordant and APOs, the prevalence of APOs in couples with GCT-discordant infection was higher than that in couples without infection or GCT-concordance, which suggests that we may need to pay more attention to the health education of GCT-discordant couples.

Some countries or regions have issued guidelines for CT infection screening (12, 14, 15), however, except for the US, many countries or regions have not carried out universal GCT infection screening for pregnant females. Our data shows that 75.38% of the male patients' partners were positive, meaning that 0.88% of wives would be infected due to male GCT-discordance. In addition, about 70% of females had no apparent symptoms after GCT infection (12). This makes many females unable to get a timely diagnosis and treatment after GCT infection, and it may lead to the occurrence of GCT sequelae and the increase of APOs.

Our study has several limitations. Firstly, we failed to collect other factors related to APOs, such as socioeconomic factors, other infection factors, drug use, etc. These factors would confuse the association between GCT infection and APOs. Secondly, the time between the occurrence of CT detection and the emergence of APOs was at least 10 months. Other events affected APOs, untreated GCT infection resolved spontaneously (37), and GCT reinfection occurred in this period, all of these conditions would affect the results. Thirdly, we did not know the CT genotypes of GCT concordant and discordant couples, as inconsistent genotypes in GCT-concordant meant both husband and wife had multiple sexual partners, it would increase reinfection risk. However, Schillinger et al. showed that 92.6% of concordant couples infected with CT showed the same genotype (34), which may occur in this study.

In conclusion, although only women's age was found to be associated with APOs, the prevalence of APOs in female and male GCT-discordant couples is higher than that in uninfected or GCT-concordant couples, especially in female, which indicates that more attention should be paid to the health education of this group in follow-ups.

## Data availability statement

The datasets presented in this article are not readily available because the datasets used and/or analyzed during the current study are available from the corresponding author on reasonable request. Requests to access the datasets should be directed to ZL, paulluo9909@163.com.

## Ethics statement

The studies involving human participants were reviewed and approved by the Ethical Committee of the Nanshan Center for Chronic Disease Control (Approved No. LL20170017). The patients/participants provided their written informed consent to participate in this study.

## Author contributions

Conceptualization, methodology, project administration, and resources: SS, LZ, QW, LT, YD, LL, HY, BL, and ZL. Data curation: SS, LZ, QW, and LT. Formal analysis: SS, HY, BL, and ZL. Investigation: SS, LZ, QW, LT, YD, LL, and HY. Software: SS and QW. Writing original draft: SS. Writing review and editing: SS, BL, and ZL. All authors contributed to the article and approved the submitted version.

## References

[1] Tang W Mao J Li KT Walker JS Chou R Fu R. Pregnancy and fertility-related adverse outcomes associated with Chlamydia trachomatis infection: a global systematic review and meta-analysis. Sex Transm Infect. (2020) 96:322–9. 10.1136/sextrans-2019-053999 31836678 PMC7292777

[2] Mwaniki MK Atieno M Lawn JE Newton CR. Long-term neurodevelopmental outcomes after intrauterine and neonatal insults: a systematic review. Lancet. (2012) 379:445–52. 10.1016/S0140-6736(11)61577-8 22244654 PMC3273721

[3] Black RE. Global prevalence of small for gestational age births. Nestle Nutr Inst Workshop Ser. (2015) 81:1–7. 10.1159/000365790 26111558

[4] Hogan DG. regional, and national causes of child mortality in 2000–13, with projections to inform post-2015 priorities: an updated systematic analysis. Lancet. (2015) 385:430–40. 10.1016/S0140-6736(14)61698-6 25280870

[5] Rowley J Vander Hoorn S Korenromp E Low N Unemo M Abu-Raddad LJ. Chlamydia, gonorrhoea, trichomoniasis and syphilis: global prevalence and incidence estimates. Bull World Health Organ. (2019) 97:548–62. 10.2471/BLT.18.228486 31384073 PMC6653813

[6] Johnson HL Ghanem KG Zenilman JM Erbelding EJ. Sexually transmitted infections and adverse pregnancy outcomes among women attending inner city public sexually transmitted diseases clinics. Sex Transm Dis. (2011) 38:167–71. 10.1097/OLQ.0b013e3181f2e85f 20852454

[7] Bagheri S Roghanian R Golbang N Golbang P Esfahani MH. Molecular evidence of chlamydia trachomatis infection and its relation to miscarriage. Int J Fertil Steril. (2018) 12:152–6. 10.22074/ijfs.2018.5184 29707933 PMC5936614

[8] Baud D Goy G Jaton K Osterheld MC Blumer S Borel N. Role of Chlamydia trachomatis in miscarriage. Emerg Infect Dis. (2011) 17:1630–5. 10.3201/eid1709.100865 21888787 PMC3322049

[9] Olson-Chen C Balaram K Hackney DN. Chlamydia trachomatis and adverse pregnancy outcomes: meta-analysis of patients with and without infection. Matern Child Health J. (2018) 22:812–21. 10.1007/s10995-018-2451-z 29417367

[10] Olaleye AO Babah OA Osuagwu CS. Sexually transmitted infections in pregnancy - An update on Chlamydia trachomatis and Neisseria gonorrhoeae. Eur J Obstet Gynecol Reprod Biol. (2020) 255:1–12. 10.1016/j.ejogrb.2020.10.002 33059307

[11] Folger AT. Maternal Chlamydia trachomatis infections and preterm birth:the impact of early detection and eradication during pregnancy. Matern Child Health J. (2014) 18:1795–802. 10.1007/s10995-013-1423-6 24337865

[12] Lanjouw E Ouburg S De Vries HJ Stary A Radcliffe K Unemo M. Background review for the 2015 European guideline on the management of Chlamydia trachomatis infections. Int J STD AIDS. (2015) 27:333–48. 10.1177/0956462415618838 26608577

[13] Ong JJ Chen M Hocking J Fairley CK Carter R Bulfone L. Chlamydia screening for pregnant women aged 16-25 years attending an antenatal service: a cost-effectiveness study. Bjog. (2016) 123:1194–202. 10.1111/1471-0528.13567 26307516

[14] MacDonald NT. Canadian guidelines on sexually transmitted infections. Cmaj. (2007) 176:175–6. 10.1503/cmaj.061616 17224598 PMC1764798

[15] Workowski KA Bachmann LH Chan PA Johnston CM Muzny CA Park I. Sexually transmitted infections treatment guidelines. MMWR Recomm Rep. (2021) 70:1–187. 10.15585/mmwr.rr7004a1 34292926 PMC8344968

[16] Gutierrez-Trujillo RP. concordance and reproductive sequelae after Chlamydia trachomatis infection in Mexican infertile couples. Andrologia. (2020) 52:e13772. 10.1111/and.13772 32722871

[17] Wei Y Xu Q Yang H Yang Y Wang L Chen H. Preconception diabetes mellitus and adverse pregnancy outcomes in over 64 million women: a population-based cohort study in China. PLoS Med. (2019) 16:e1002926. 10.1371/journal.pmed.1002926 31574092 PMC6771981

[18] Colonna C Steelman M. Amsel Criteria, in StatPearls, StatPearls Publishing Copyright *2022*. Treasure Island, FL: StatPearls Publishing LLC (2022).

[19] Mengistie Z Woldeamanuel Y Asrat D Yigeremu M. Comparison of clinical and gram stain diagnosis methods of bacterial vaginosis among pregnant women in ethiopia. J Clin Diagn Res. (2013) 7:2701–3. 10.7860/JCDR/2013/5872.3736 24551617 PMC3919279

[20] Parra-Sánchez M Palomares JC Bernal S González MT Sivianes N Pérez L. Evaluation of the cobas 4800 CT/NG Test for detecting Chlamydia trachomatis and Neisseria gonorrhoeae DNA in urogenital swabs and urine specimens. Diagn Microbiol Infect Dis. (2012) 74:338–42. 10.1016/j.diagmicrobio.2012.08.004 22995365

[21] Wang LL Bai RH Liu Q Zhang Q Dang SN Mi BB et al. Epidemiological study on adverse pregnancy outcomes in Shaanxi province. Chin J Epidemiol. (2016) 37:1379-82. 10.3760/cma.j.issn.0254-6450.2016.10.013 27765130

[22] Adib-Rad H Basirat Z Faramarzi M Mostafazadeh A Bijani A. Psychological distress in women with recurrent spontaneous abortion: a case-control study. Turk J Obstet Gynecol. (2019) 16:151–7. 10.4274/tjod.galenos.2019.88899 31673466 PMC6792057

[23] Pei L Kang Y Zhao Y Cheng Y Yan H. Changes in socioeconomic inequality of low birth weight and macrosomia in Shaanxi Province of Northwest China 2010–2013: a cross-sectional study. Medicine. (2016) 95:e2471. 10.1097/MD.0000000000002471 26844457 PMC4748874

[24] Guo T Wang Y Zhang H Zhang Y Zhao J Wang Y. The association between ambient temperature and the risk of preterm birth in China. Sci Total Environ. (2018) 613:439–46. 10.1016/j.scitotenv.2017.09.104 28918275

[25] Lin S Zhang Y Li J Wu J Pei L. Trends of adverse pregnancy outcomes in a high prevalence region of birth defects - Shanxi Province, China 2007–2019. China CDC Wkly. (2021) 3:661–4. 10.46234/ccdcw2021.167 34594963 PMC8392908

[26] Knop MR Geng TT Gorny AW Gorny AW Ding R Li C. Birth weight and risk of type 2 diabetes mellitus, cardiovascular disease, and hypertension in adults: a meta analysis of 7,646,267 participants from 135 studies. J Am Heart Assoc. (2018) 7:e008870. 10.1161/JAHA.118.008870 30486715 PMC6405546

[27] Sadowska M Sarecka-Hujar B Kopyta I. Cerebral palsy: current opinions on definition, epidemiology, risk factors, classification and treatment options. Neuropsychiatr Dis Treat. (2020) 16:1505–18. 10.2147/NDT.S235165 32606703 PMC7297454

[28] Frederiksen LE Ernst A Brix N Lauridsen LL Roos L Ramlau-Hansen CH. Risk of adverse pregnancy outcomes at advanced maternal age. Obstet Gynecol. (2018) 131:457–63. 10.1097/AOG.0000000000002504 29420406

[29] Waldenström U Cnattingius S Vixner L Norman M. Advanced maternal age increases the risk of very preterm birth, irrespective of parity: a population-based register study. Bjog. (2017) 124:1235–44. 10.1111/1471-0528.14368 27770495

[30] Fitzpatrick KE Tuffnell D Kurinczuk JJ Knight M. Pregnancy at very advanced maternal age: a UK population-based cohort study. Bjog. (2017) 124:1097–106. 10.1111/1471-0528.14269 27581343 PMC5484369

[31] Lean SC Derricott H Jones RL Heazell AE. Advanced maternal age and adverse pregnancy outcomes: a systematic review and meta-analysis. PLoS ONE. (2017) 12:e0186287. 10.1371/journal.pone.0186287 29040334 PMC5645107

[32] Luo ZZ Li W Wu QH Zhang L Tian LS Liu LL. Population-based study of chlamydial and gonococcal infections among women in Shenzhen, China: implications for programme planning. PLoS One. (2018) 13:e0196516. 10.1371/journal.pone.0196516 29715319 PMC5929501

[33] Vercruysse J Mekasha S Stropp LM Moroney J He X Liang Y. Chlamydia trachomatis infection, when treated during pregnancy, is not associated with preterm birth in an urban safety-net hospital. Infect Dis Obstet Gynecol. (2020) 2020:8890619. 10.1155/2020/8890619 33082702 PMC7556048

[34] Schillinger JA Katz BP Markowitz LE Braslins PG Shrier LA Madico G. Genotype-specific concordance of chlamydia trachomatis genital infection within heterosexual partnerships. Sex Transm Dis. (2016) 43:741–9. 10.1097/OLQ.0000000000000525 27835626 PMC5113142

[35] Quinn TC Gaydos C Shepherd M Bobo L Hook EW Viscidi R. Epidemiologic and microbiologic correlates of Chlamydia trachomatis infection in sexual partnerships. JAMA. (1996) 276:1737–42. 10.1001/jama.1996.03540210045032 8940322

[36] Guerra-Infante FM Tapia-Yáñez JR López-Hurtado M Flores-Medina S Díaz-García FJ. Chlamydia trachomatis infection in men and its association with gynecologic alterations in their sexual partners. Rev Invest Clin. (2005) 57:406–14. 16187700

[37] Geisler WM Lensing SY Press CG Hook III EW. Spontaneous resolution of genital Chlamydia trachomatis infection in women and protection from reinfection. J Infect Dis. (2013) 207:1850–6. 10.1093/infdis/jit094 23470847 PMC3654745

